# Supplementary material for: Signature of anyonic statistics in the integer quantum Hall regime
Source: Nat Commun. 2024 Aug 3;15:6578. doi: 10.1038/s41467-024-50820-0 (PMC11297956; doi:10.1038/s41467-024-50820-0)
Supplement: Supplementary file 1 — Supplementary Information [file 41467_2024_50820_MOESM1_ESM.pdf]

Supplementary Information for
Signature of anyonic statistics in the integer quantum Hall regime

P. Glidic,[1] I. Petkovic,[1] C. Piquard,[1] A. Aassime,[1] A. Cavanna,[1] Y. Jin,[1]
U. Gennser,[1] C. Mora,[2] D. Kovrizhin,[3] A. Anthore,[1,4] and F. Pierre[1]

[1] *Université Paris-Saclay, CNRS, Centre de Nanosciences et de Nanotechnologies, 91120, Palaiseau, France*
[2] *Université Paris Cité, CNRS, Laboratoire Matériaux et Phénomènes Quantiques, F-75013 Paris, France*
[3] *CY Cergy Paris Université, CNRS, Laboratoire de Physique Théorique et Modélisation, Cergy-Pontoise, F-95302, France*
[4] *Université Paris Cité, CNRS, Centre de Nanosciences et de Nanotechnologies, F-91120, Palaiseau, France*

Note that the Supplementary Information refers to its own set of references, separate from those in the main manuscript.

I. NON-PERTURBATIVE THEORY

A. Introduction

In this Section we outline the theoretical description of the electron collider using a model which can be solved via refermionization techniques. We also present the results for the asymptotics of the noise in the small tunneling limit.

The notation in the ‘Non-perturbative theory’ Section is different from the rest of the paper in order to keep the correspondence with the previous work Ref. 1 and with an upcoming theoretical publication providing further details (D. Kovrizhin, in preparation).

The notation conversion is the following :

**This section**	**Elsewhere**
T_1	τ_{s}
T_2	τ_{s}
T_S	τ_{c}
bias V	bias V_{s}
spin up (↑) channel	inner channel
spin down (↓) channel	outer channel
1’ channels (Fig. **1**)	region biased by V_3 (Fig. **1**c Main text)
2’ channels (Fig. **1**)	region biased by V_1 (Fig. **1**c Main text)
1 channels (Fig. **1**)	region biased by V_2 (Fig. **1**c Main text)
2 channels (Fig. **1**)	region biased by V_4 (Fig. **1**c Main text)

Supplementary Table I. Notation correspondence between this Section and the rest of the paper.

The schematics of the model is shown in Supplementary Fig. **1** for the noise measurement between channels $1_\downarrow$ and $2'_\downarrow$. In the Main text this is outlined as the default configuration, i.e., injection and measurement in the outer channel. Other configurations can be calculated in a similar manner, and the theory will be detailed in the above mentioned upcoming article.

Let us consider a system with four edge states $1, 1', 2, 2'$, each of which is carrying two co-propagating edge channels $(\uparrow, \downarrow)$, where the arrows denote the spin. The biasing scheme of biasing source quantum point contacts with 0 and eV in Supplementary Fig. **1** is equivalent to biasing with $-eV/2$ and $+eV/2$ (used in the experiment) due to invariance of the observables under a global potential shift.

We model the non-interacting edge channels by the free-fermion Hamiltonian with linear dispersion (assuming the same Fermi-velocity v_F in every channel). This is a standard description of edge states at integer filling factors. In addition, we assume that electrons on each edge interact via short-range interactions with strength g which has the dimension of a velocity. This model of interactions provides a good description of previous experiments at filling factor $\nu = 2$, where interactions are usually sufficiently strong to overcome any asymmetry between channels[1–3]. We note that in the experimental setup the long-range Coulomb interactions are expected to be screened by the metallic gates over a typical length scale of a few hundred nanometers of the order of the distance between 2DEG and nearest gate.

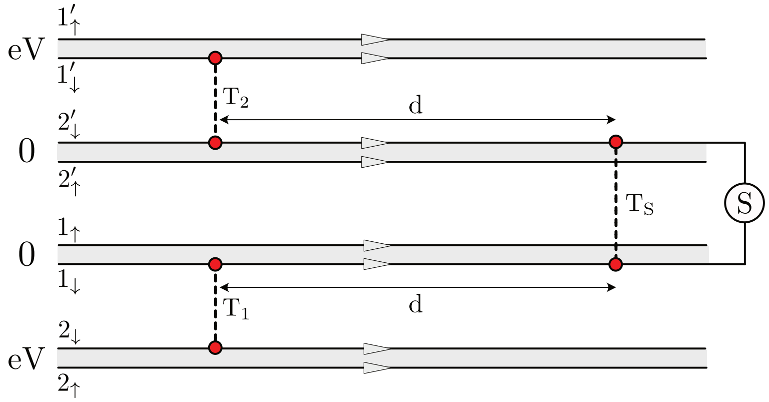

Supplementary Figure 1. Schematic of the model of the electron collider at $\nu = 2$ with four chiral quantum Hall edges ($1, 1', 2, 2'$), each carrying two edge channels ($\uparrow, \downarrow$). The channels $1_\downarrow$ and $2_\downarrow$ are coupled via the source QPC$_1$ positioned at $x = 0$ and characterized by the tunneling probability T_1. Similarly the edge states $1'_\downarrow$ and $2'_\downarrow$ are coupled by QPC$_2$ with tunneling probability T_2. At $x = d$ downstream from the sources, the channels $2'_\downarrow$ and $1_\downarrow$ are coupled by the analyzer QPC$_S$ of tunneling probability T_S. The considered interaction is short-range, acting between the channels which propagate along the same quantum Hall edge, separated by a narrow strip shown in grey. The two top channels ($1'_{\uparrow,\downarrow}$) and the two bottom channels ($2_{\uparrow,\downarrow}$) are biased with the chemical potentials eV, whereas the four channels in the middle ($1_{\uparrow,\downarrow}$, $2'_{\uparrow,\downarrow}$) are grounded. The current correlations are measured after QPC$_S$. Solvable variants of the model include using the analyzer QPC$_S$ to couple the other channels ($2'_\uparrow$ with $1_\uparrow$) for the data-theory comparison in, e.g., Fig. **4**b of the Main text, or applying the same voltage V_p to the four bottom channels ($1_{\uparrow,\downarrow}$, $2_{\uparrow,\downarrow}$) for the data-theory comparison on electron distribution spectroscopy (see corresponding schematic in Supplementary Fig. **2**).

The Hamiltonian of the system shown in Supplementary Fig. **1** reads:

$$\hat{H} = -i\hbar v_F \sum_{\eta=1,1',2,2',s=\uparrow,\downarrow} \int_{-\infty}^{\infty} dx\ \hat{\Psi}^\dagger_{\eta s}(x)\partial_x\hat{\Psi}_{\eta s}(x) + 2\pi\hbar g \sum_{\eta=1,1',2,2'} \int_{-\infty}^{\infty} \hat{\rho}_{\eta\uparrow}(x)\hat{\rho}_{\eta\downarrow}(x)dx + \left(v_1\hat{\Psi}^\dagger_{1\downarrow}(0)\hat{\Psi}_{2\downarrow}(0) + v_2\hat{\Psi}^\dagger_{1'\downarrow}(0)\hat{\Psi}_{2'\downarrow}(0) + v_S\hat{\Psi}^\dagger_{2'\downarrow}(d)\hat{\Psi}_{1\downarrow}(d) + \text{h.c.}\right). \quad (1)$$

Here d is the distance between the source QPC$_{1,2}$ and the analyzer QPC$_S$. $\hat{\Psi}_{\eta s}(x)$ are fermion annihilation operators and $\hat{\rho}_{\eta s}(x)$ are fermion density operators on the corresponding edge channels (ηs). $v_{1,2,S}$ are the tunneling amplitudes connected to the transmission probabilities $T_{1,2,S}$ across QPC$_{1,2,S}$ ($T_{1,2,S} = \sin^2(|v_{1,2,S}|/\hbar v_F)$ in the non-interacting case). See Ref. 4 for details of the refermionization approach with the same notations as in this Section.

The zero-frequency cross-correlation noise $S_{1\downarrow 2'\downarrow}$ between the channels $1_\downarrow$ and $2'_\downarrow$ outgoing from the analyzer QPC$_S$, at the position $x \geqslant d$ (see Supplementary Fig. **1**), is obtained from:

$$S_{1\downarrow 2'\downarrow}(V) = 2\int_{-\infty}^{\infty} dt\ \langle \delta\hat{I}_{1\downarrow}(x,t)\delta\hat{I}_{2'\downarrow}(x,0)\rangle, \quad (2)$$

with $\delta\hat{I}_{\eta s}(x,t)$ written in terms of the current operators $I^H_{\eta s}(x,t)$ in the Heisenberg representation as:

$$\delta\hat{I}_{\eta s}(x,t) = \hat{I}^H_{\eta s}(x,t) - \langle\hat{I}^H_{\eta s}(x,t)\rangle. \quad (3)$$

The current operators in the interaction representation, where the tunneling plays the role of the ‘interaction’, are given by the following expression

$$\hat{I}_{\eta s}(x,t) = -ev_F\hat{\rho}_{\eta s}(x,t). \quad (4)$$

The model described by the Hamiltonian given in Eq. (1) can be solved for arbitrary interaction strengths using the refermionization approach[4], as sketched in the next subsections (further details in D. Kovrizhin, in preparation). As a check, note that when applying this refermionization approach to the non-interacting case $g = 0$ at $T = 0$, we recover the same expression derived in the free electron scattering model:

$$S^{(0)}_{1\downarrow 2'\downarrow}(V) = -2\left(\frac{e^2}{2\pi\hbar}\right)T_S R_S (T_1 - T_2)^2|eV|, \quad (5)$$

where $R_{1,2,S} \equiv 1 - T_{1,2,S}$ are the reflection probabilities. Note also that the noise in the non-interacting case is independent of the distance d, and is always zero in the case of equal tunneling probabilities on the source QPC$_{1,2}$ ($T_1 = T_2$).

B. Bosonization

We would like to bosonize the Hamiltonian in Eq. (1). After introducing bosonic operators $\hat{\phi}_{\eta s}(x)$, Klein factors $\hat{F}_{\eta s}$, and number operators $\hat{N}_{\eta s}$ in the Schrödinger representation, as well as a short-distance cutoff a, we can write the fermion operators in the bosonic form:

$$\hat{\Psi}_{\eta s}(x) = \frac{1}{\sqrt{2\pi a}} \hat{F}_{\eta s} e^{i\frac{2\pi}{L}\hat{N}_{\eta s}x} e^{-i\hat{\phi}_{\eta s}(x)}. \tag{6}$$

The operators obey the following commutation relations:

$$[\hat{\phi}_{\eta s}(x), \partial_y \hat{\phi}_{\eta' s'}(y)] = -2\pi i \delta_{\eta\eta'}\delta_{ss'}\delta(x-y), \quad \{\hat{F}^\dagger_{\eta s}, \hat{F}_{\eta' s'}\} = 2\delta_{\eta\eta'}\delta_{ss'}, \quad [\hat{N}_{\eta s}, \hat{F}_{\eta' s'}] = -\delta_{\eta\eta'}\delta_{ss'}\hat{F}_{\eta s}, \tag{7}$$

and the density operator is written in terms of the bosonic fields as

$$\hat{\rho}_{\eta s}(x) = \frac{\hat{N}_{\eta s}}{L} - \frac{1}{2\pi}\partial_x \hat{\phi}_{\eta s}(x). \tag{8}$$

In this bosonic representation, the Hamiltonian in Eq. (1) can be written as:

$$\begin{aligned}\hat{H} = \frac{\hbar v_F}{2}\sum_{\eta s}\int \frac{dx}{2\pi}(\partial_x\hat{\phi}_{\eta s})^2 + g\hbar\sum_{\eta}\int\frac{dx}{2\pi}\partial_x\hat{\phi}_{\eta\uparrow}\partial_x\hat{\phi}_{\eta\downarrow} + \frac{2\pi g\hbar}{L}\sum_{\eta}\hat{N}_{\eta\uparrow}\hat{N}_{\eta\downarrow} + \frac{2\pi}{L}\frac{\hbar v_F}{2}\sum_{\eta s}\hat{N}_{\eta s}(\hat{N}_{\eta s}+1) \\ + \frac{1}{2\pi a}\left(v_1 \hat{F}^\dagger_{1\downarrow}\hat{F}_{2\downarrow}e^{i(\hat{\phi}_{1\downarrow}(0)-\hat{\phi}_{2\downarrow}(0))} + v_2 \hat{F}^\dagger_{1'\downarrow}\hat{F}_{2'\downarrow}e^{i(\hat{\phi}_{1'\downarrow}(0)-\hat{\phi}_{2'\downarrow}(0))} + v_S \hat{F}^\dagger_{2'\downarrow}\hat{F}_{1\downarrow}e^{i(\hat{\phi}_{2'\downarrow}(0)-\hat{\phi}_{1\downarrow}(0))} + \text{h.c.}\right).\end{aligned} \tag{9}$$

C. Refermionization

Here we show how to refermionize the Hamiltonian in Eq. (9), which allows one to obtain the exact expressions for the noise in the presence of interactions. To express the current correlators we will use refermionization, which permits us to map our model onto a system of non-interacting fermions for each QPC separately. This does not mean that there is no dependence of the noise on the interactions because the transformations between the new fields and the original fields will generate an interaction-dependent contribution to the noise.

We start with the refermionization of the four channels $(1_\uparrow, 1_\downarrow, 2'_\downarrow, 2'_\uparrow)$. For this purpose we first introduce new bosonic operators $\tilde{\chi}_{S+}(x), \tilde{\chi}_{A-}(x), \tilde{\chi}_{A+}(x), \tilde{\chi}_{S-}(x)$, which are related to the original bosonic operators $\phi_{\eta s}$ via the transformation $\tilde{\chi}^T = U\phi^T$ where U is the following 4×4 matrix:

$$U = \frac{1}{2}\begin{pmatrix} 1 & 1 & 1 & 1 \\ 1 & -1 & 1 & -1 \\ 1 & 1 & -1 & -1 \\ 1 & -1 & -1 & 1 \end{pmatrix}. \tag{10}$$

Note that the dispersions of the bosons corresponding to $\tilde{\chi}_{A,S+}$ and $\tilde{\chi}_{A,S-}$ are given by the velocities $v_+ = v_F + g$ and $v_- = v_F - g$, respectively.

We would like to evaluate current correlation functions at non-equal times for the channels $1_\downarrow$ and $2'_\downarrow$ at some position after the QPC$_S$. For that we need to have expressions for the currents in these channels in terms of refermionized operators. The currents in terms of the original fermions can be obtained from the Heisenberg equations of motion for the density operators (in the interaction representation, where the tunneling is treated as an interaction term in the Hamiltonian):

$$\partial_t\hat{\rho}_{1\downarrow}(x,t) = -\partial_x(v_F\hat{\rho}_{1\downarrow}(x,t) + g\hat{\rho}_{1\uparrow}(x,t)), \quad \partial_t\hat{\rho}_{2'\downarrow}(x,t) = -\partial_x(v_F\hat{\rho}_{2'\downarrow}(x,t) + g\hat{\rho}_{2'\uparrow}(x,t)). \tag{11}$$

The corresponding currents are expressed in terms of the original fermions in the interaction representation as

$$\hat{I}_{1\downarrow}(x,t) = -e(v_F\hat{\rho}_{1\downarrow}(x,t) + g\hat{\rho}_{1\uparrow}(x,t)), \quad \hat{I}_{2'\downarrow}(x,t) = -e(v_F\hat{\rho}_{2'\downarrow}(x,t) + g\hat{\rho}_{2'\uparrow}(x,t)), \tag{12}$$

with $\hat{\rho}_{\eta s}$ given by Eq. (8). In the following we will omit the number operators $\hat{N}_{\eta s}$ appearing in Eq. (8) in order to simplify the notations. Since they transform in the same way as the fields under linear transformation with the matrix U, we will be able to restore them at the end.

Using the inverse transformation U^{-1} ($U^{-1} = U$) we can write the currents at position d in terms of the transformed density operators $\tilde{\rho}_{S,A,\pm}$, which are related to the $\tilde{\chi}$ fields via an equation analogous to Eq. (8), as

$$\hat{I}_{1\downarrow}(d,t) = -e\frac{1}{2}\left(v_+(\tilde{\rho}_{S+}(d,t) + \tilde{\rho}_{A+}(d,t)) - v_-(\tilde{\rho}_{A-}(d,t) + \tilde{\rho}_{S-}(d,t))\right), \tag{13}$$

$$\hat{I}_{2'\downarrow}(d,t) = -e\frac{1}{2}\left(v_+(\tilde{\rho}_{S+}(d,t) - \tilde{\rho}_{A+}(d,t)) + v_-(\tilde{\rho}_{A-}(d,t) - \tilde{\rho}_{S-}(d,t))\right). \tag{14}$$

We can rewrite these expressions in the more convenient form

$$\hat{I}_{1\downarrow}(d,t) = \frac{e}{2}(\hat{I}_0(d,t) + \hat{I}_1(d,t)), \quad \hat{I}_{2'\downarrow} = \frac{e}{2}(\hat{I}_0(d,t) - \hat{I}_1(d,t)), \tag{15}$$

where we have defined

$$\hat{I}_0(d,t) \equiv -v_+\tilde{\rho}_{S+}(d,t) + v_-\tilde{\rho}_{S-}(d,t), \quad \hat{I}_1(d,t) \equiv -v_+\tilde{\rho}_{A+}(d,t) + v_-\tilde{\rho}_{A-}(d,t). \tag{16}$$

We can now proceed with the calculations of the noise, obtained by integrating in time the following current correlator

$$\langle \hat{I}_{1\downarrow}(d,t_1)\hat{I}_{2'\downarrow}(d,t_2)\rangle = \langle \hat{I}_0(d,t_1)\hat{I}_0(d,t_2)\rangle - \langle \hat{I}_1(d,t_1)\hat{I}_1(d,t_2)\rangle - \langle \hat{I}_0(d,t_1)\hat{I}_1(d,t_2)\rangle + \langle \hat{I}_1(d,t_1)\hat{I}_0(d,t_2)\rangle. \tag{17}$$

We now proceed with the above correlator. Whereas the last two terms cancel out as $\hat{I}_0(d,t_{1,2})$ and $\hat{I}_1(d,t_{2,1})$ commute, it is not the case of the first two terms. After refermionization, one can write the Hamiltonian for the four channels coupled by QPC$_S$ in terms of free fermions with the standard tunneling term $H^{\rm ref}_{T_S} = \tilde{v}_S\tilde{\Psi}^\dagger_{A_+}(d)\tilde{\Psi}_{A_-}(d) + \text{h.c.}$, with $\tilde{v}_S$ being the renormalized tunneling strength[4] directly related to the transmission probability T_S measured in the experiment. Note that $H^{\rm ref}_{T_S}$ does not affect the fields $S_\pm$. The fermion operators $\tilde{\Psi}_{A_\pm}$ are transformed by QPC$_S$ as

$$\begin{aligned}\tilde{\Psi}_{A_+}(d^+,t) &= r_S\tilde{\Psi}_{A_+}(d^-,t) - it_S\tilde{\Psi}_{A_-}(d^-,t),\\ \tilde{\Psi}_{A_-}(d^+,t) &= -it_S\tilde{\Psi}_{A_+}(d^-,t) + r_S\tilde{\Psi}_{A_+}(d^-,t),\end{aligned} \tag{18}$$

denoting the transmission and reflection amplitudes as t_S and r_S ($|t_S|^2 \equiv T_S$, $|r_S|^2 \equiv R_S \equiv 1 - T_S$), and where d^+ and d^- are the positions just after and just before QPC$_S$, respectively.

Let us start with $\langle \hat{I}_0(d,t_1)\hat{I}_0(d,t_2)\rangle$. Because the operator $\hat{I}_0$ does not transform under the action of $H^{\rm ref}_{T_S}$, we can write the current $\hat{I}_0$ in terms of the original bosonic fields $\hat{\phi}$:

$$\hat{I}_0(d,t) = -\frac{v_+}{2}\left(\hat{\phi}_{1\uparrow}(d,t) + \hat{\phi}_{1\downarrow}(d,t) + \hat{\phi}_{2'\downarrow}(d,t) + \hat{\phi}_{2'\uparrow}(d,t)\right) + \frac{v_-}{2}\left(\hat{\phi}_{1\uparrow}(d,t) - \hat{\phi}_{1\downarrow}(d,t) - \hat{\phi}_{2'\downarrow}(d,t) + \hat{\phi}_{2'\uparrow}(d,t)\right). \tag{19}$$

We note that the operators on the right hand side are given in the Heisenberg representation, which includes the interactions and the tunneling at both QPC$_1$ and QPC$_2$.

We now need to refermionize the subsystems connected by QPC$_1$ and QPC$_2$ (e.g., we refermionize separately channels with primed indices, and channels with unprimed indices). In order to do that we introduce operators $\hat{\chi}(x)$ related to transformations of the bottom four channels and operators $\hat{\chi}'(x)$ related to top four channels

$$(\hat{\chi}_{S+}(x), \hat{\chi}_{A-}(x), \hat{\chi}_{A+}(x), \hat{\chi}_{S-}(x))^T = U(\hat{\phi}_{1\uparrow}(x), \hat{\phi}_{1\downarrow}(x), \hat{\phi}_{2\downarrow}(x), \hat{\phi}_{2\uparrow}(x))^T \tag{20}$$

$$(\hat{\chi}'_{S+}(x), \hat{\chi}'_{A-}(x), \hat{\chi}'_{A+}(x), \hat{\chi}'_{S-}(x))^T = U(\hat{\phi}_{1'\uparrow}(x), \hat{\phi}_{1'\downarrow}(x), \hat{\phi}_{2'\downarrow}(x), \hat{\phi}_{2'\uparrow}(x))^T. \tag{21}$$

Using these transformations we can write the current operator $\hat{I}_0(d,t)$ as

$$\hat{I}_0(d,t) = -\frac{v_+}{2}\left(\hat{\rho}_{S_+}(d,t) + \hat{\rho}_{A_+}(d,t) + \hat{\rho}'_{S_+}(d,t) - \hat{\rho}'_{A_+}(d,t)\right) + \frac{v_-}{2}\left(\hat{\rho}_{A_-}(d,t) + \hat{\rho}_{S_-}(d,t) - \hat{\rho}'_{A_-}(d,t) + \hat{\rho}'_{S_-}(d,t)\right). \tag{22}$$

We note that there is no coherence between primed and unprimed terms as well as between S_+, S_- terms because they are not connected by refermionized QPC$_{1,2}$ (as with QPC$_S$, only A_+, A_- and A'_+, A'_- channels are connected by QPC$_{1,2}$ correspondingly).

The operators in the current (taken after $\mathrm{QPC}_{1,2}$) should be related to the operators before $\mathrm{QPC}_{1,2}$. For this purpose, we use

$$\hat{\Psi}^\dagger_{A_-}(0^+,\tau)\hat{\Psi}_{A_-}(0^+,\tau) = \left(+it_1\hat{\Psi}^\dagger_{A_+}(0^-,\tau) + r_1\hat{\Psi}^\dagger_{A_-}(0^-,\tau)\right)\left(-it_1\hat{\Psi}_{A_+}(0^-,\tau) + r_1\hat{\Psi}_{A_-}(0^-,\tau)\right), \tag{23}$$

and the similar transformation of A_+ operators at 0^+. These transformations allow us to write the current correlators (noting that the A_+ and A_- operators on the right hand side are incoherent because they are taken before QPC_1, at position 0^-). Denoting as $\delta\tau \equiv t_1 - t_2 - d/v_{\mathrm{eff}}$, where $v_{\mathrm{eff}} = (1/v_- - 1/v_+)^{-1}$ is an effective velocity, we obtain (for voltage-dependent terms)

$$\begin{aligned}\langle\langle \hat{I}_0(d,t_1)\hat{I}_0(d,t_2)\rangle\rangle = R_1T_1\left(G_{A_+}(\delta\tau)\bar{G}_{A_-}(\delta\tau) + G_{A_-}(\delta\tau)\bar{G}_{A_+}(\delta\tau)\right)&\\ + R_2T_2\left(G_{A'_+}(\delta\tau)\bar{G}_{A'_-}(\delta\tau) + G_{A'_-}(\delta\tau)\bar{G}_{A'_+}(\delta\tau)\right),&\end{aligned} \tag{24}$$

where double brackets denote normal-ordering of the current operators, and

$$G_{S,A,\pm}(\delta\tau) = \frac{i}{2\pi}\frac{e^{\frac{i}{\hbar}\mu_{S,A,\pm}\delta\tau}}{\delta\tau - ia} \tag{25}$$

are free-fermion Green functions. In order to evaluate this correlator we need to know the chemical potentials of the channels in the refermionized representation. These chemical potentials follow the refermionization prescription (and can be obtained using matrix U), which gives for this voltage setup (the distribution function setting would correspond to different values, see Ref. 1)

$$\begin{aligned}&\mu_{A_+} = -eV,\ \mu_{A_-} = 0,\ \mu_{S_+} = eV,\ \mu_{S_-} = 0,\\ &\mu_{A'_+} = eV,\ \mu_{A'_-} = 0,\ \mu_{S'_+} = eV,\ \mu_{S'_-} = 0.\end{aligned} \tag{26}$$

This gives:

$$\langle\langle \hat{I}_0(d,t_1)\hat{I}_0(d,t_2)\rangle\rangle = -\frac{2}{(2\pi)^2}\frac{1}{\delta\tau^2}(R_1T_1 + R_2T_2)\cos(eV\delta\tau/\hbar), \tag{27}$$

where we note that the result is independent of the position d.

Similarly, after some algebra, we find for the correlator of I_1 operators the following expression (shown here again for voltage-dependent terms)

$$\begin{aligned}\langle\langle \hat{I}_1(d,t_1)\hat{I}_1(d,t_2)\rangle\rangle = &+(R_s - T_s)^2R_1T_1\left(G_{A_-}(\delta\tau)\bar{G}_{A_+}(\delta\tau) + G_{A_+}(\delta\tau)\bar{G}_{A_-}(\delta\tau)\right)\\ &+ (R_s - T_s)^2R_2T_2\left(G_{A'_-}(\delta\tau)\bar{G}_{A'_+}(\delta\tau) + G_{A'_+}(\delta\tau)\bar{G}_{A'_-}(\delta\tau)\right)\\ &+ 4R_sT_s\tilde{v}^2\left(G_{1\downarrow}(d,t_1-t_2)\bar{G}_{2'\downarrow}(d,t_1-t_2) + G_{2'\downarrow}(d,t_1-t_2)\bar{G}_{1\downarrow}(d,t_1-t_2)\right),\end{aligned} \tag{28}$$

where we have introduced the interacting Green functions defined as

$$G_{1\downarrow}(d,t_1-t_2) = \langle\hat{\Psi}^\dagger_{1\downarrow}(d,t_1)\hat{\Psi}_{1\downarrow}(d,t_2)\rangle,\quad \bar{G}_{2'\downarrow}(d,t_1-t_2) = \langle\hat{\Psi}_{2'\downarrow}(d,t_1)\hat{\Psi}^\dagger_{2'\downarrow}(d,t_2)\rangle. \tag{29}$$

The first two terms in Eq. (28) involve solely the non-interacting Green functions ($G_{A_\pm}$,$G_{A'_\pm}$) and can be expressed using the values of the chemical potentials given Eq. (26) as $2(R_S - T_S)^2(R_1T_1 + R_2T_2)\cos(eV\delta\tau/\hbar)/\delta\tau^2$, in a similar form as Eq. (27). This gives:

$$\begin{aligned}\langle\langle \hat{I}_1(d,t_1)\hat{I}_1(d,t_2)\rangle\rangle = &\,2(R_S - T_S)^2(R_1T_1 + R_2T_2)\cos(eV\delta\tau/\hbar)/\delta\tau^2\\ &+ 4R_ST_S\tilde{v}^2\left(G_{1\downarrow}(d,t_1-t_2)\bar{G}_{2'\downarrow}(d,t_1-t_2) + G_{2'\downarrow}(d,t_1-t_2)\bar{G}_{1\downarrow}(d,t_1-t_2)\right).\end{aligned} \tag{30}$$

D. Collider configuration

1. Zero temperature noise

Here we only present the result at zero temperature, and the finite-temperature result can be obtained in a similar way. The finite temperature version used to calculate numerically the cross-correlations shown in Figs. **3** and **4** in the Main text and Supplementary Figure **10** is provided in the subsection 'Finite temperature expressions'.

Integrating over $\delta\tau$, the $T=0$ correlators given by Eqs. (27) and (30) yield the cross-correlation noise at zero frequency as a function of the interacting Green functions $G_{1\downarrow}$ and $G_{2'\downarrow}$:

$$S_{1\downarrow 2'\downarrow}(V) = 2\frac{e^2}{2\pi\hbar}R_S T_S(R_1T_1+R_2T_2)|eV| \\ -2e^2 R_S T_S \tilde{v}^2\int_{-\infty}^{\infty} dt\left(G_{1\downarrow}(d,t)\bar{G}_{2'\downarrow}(d,t)+G_{2'\downarrow}(d,t)\bar{G}_{1\downarrow}(d,t)-(\text{same at } V=0)\right), \quad (31)$$

where $\tilde{v}=\sqrt{v_+v_-}$. Note that the noise $S_{1\downarrow 2'\downarrow}$ from Eq. (31) can be written as a sum of the non-interacting contribution $S^{(0)}_{1\downarrow 2'\downarrow}$ given in Eq. (5) and an interacting contribution $S^{(\mathrm{int})}_{1_\downarrow 2'_\downarrow}$:

$$S_{1\downarrow 2'\downarrow}(V) = S^{(0)}_{1\downarrow 2'\downarrow}(V)+S^{(\mathrm{int})}_{1\downarrow 2'\downarrow}(V), \quad (32)$$

where

$$S^{(\mathrm{int})}_{1\downarrow 2'\downarrow}(V) = 2\frac{e^2}{2\pi\hbar}R_S T_S(R_1T_2+T_1R_2)|eV| - 2e^2R_ST_S\tilde{v}^2\int_{-\infty}^{\infty}dt\left(G_{1\downarrow}(d,t)\bar{G}_{2'\downarrow}(d,t)+G_{2'\downarrow}(d,t)\bar{G}_{1\downarrow}(d,t)\right). \quad (33)$$

The interacting Green functions can be obtained from refermionization, for example we have

$$\bar{G}_{1\downarrow}(d,t) = -\frac{i}{2\pi t\tilde{v}}\exp(-\tfrac{i}{2\hbar}eVt)\bar{K}_{1\downarrow}(d,t), \quad (34)$$

where the function $\bar{K}_{1\downarrow}(d,t)$ is defined as

$$\bar{K}_{1\downarrow}(d,t) = \frac{\left\langle \mathcal{S}_1^\dagger \exp\left(-i\pi\mathcal{N}_{A_+}(d,t)+i\pi\mathcal{N}_{A_-}(d,t)\right)\mathcal{S}_1\right\rangle}{\left\langle \exp\left(-i\pi\mathcal{N}_{A_+}(d,t)+i\pi\mathcal{N}_{A_-}(d,t)\right)\right\rangle}. \quad (35)$$

Here, the averages are taken with respect to the filled Fermi seas at the corresponding chemical potentials, and the scattering matrix $\mathcal{S}_1$ corresponds to the rotation of fermions $\hat{\Psi}_{A_+}, \hat{\Psi}_{A_-}$ by QPC$_1$ in the standard way (see also Eq. (18) for QPC$_S$). We have defined the particle number operators[1,4] $\mathcal{N}_{A_\pm}(d,t)$ for the refermionized channels with velocities $v_\pm$ as

$$\mathcal{N}_{A_\pm}(d,t) = \int_{-d/v_\pm}^{t-d/v_\pm} \hat{\Psi}^\dagger_{A_\pm}(0,\tau)\hat{\Psi}_{A_\pm}(0,\tau)d\tau \quad (36)$$

where the fermion operators are given in the interaction representation with tunneling at QPC$_1$ treated as interaction. These particle number operators count the number of particles passing position $x=0$ in a time window $(-d/v_\pm, -d/v_\pm+t)$.

We note that functions K have a form similar to the full counting statistics (FCS). At large distances the two exponents are uncorrelated, and these functions can be analysed by the methods developed for the FCS. At intermediate distances we have to rely on calculations using fermionic determinants[4]. In order to numerically calculate their values, we first obtained analytically the matrix elements of the FCS exponents with respect to the filled Fermi seas in the corresponding channels with one extra particle/hole. Then, using these matrix elements in the expressions given in terms of fermionic determinants, we calculated the functions $K(d,t)$ (details will be provided in D. Kovrizhin in preparation, see also Appendix A in Ref. 4).

Similarly, the Green function for channel $2'_\downarrow$ is obtained as

$$G_{2'\downarrow}(d,t) = -\frac{i}{2\pi t\tilde{v}}\exp(\tfrac{i}{2\hbar}eVt)K_{2'\downarrow}(d,t), \quad (37)$$

where

$$K_{2'\downarrow}(d,t) = \frac{\left\langle \mathcal{S}_2^\dagger \exp\left(-i\pi\mathcal{N}_{A'_+}(d,t)+i\pi\mathcal{N}_{A'_-}(d,t)\right)\mathcal{S}_2\right\rangle}{\left\langle \exp\left(-i\pi\mathcal{N}_{A'_+}(d,t)+i\pi\mathcal{N}_{A'_-}(d,t)\right)\right\rangle}. \quad (38)$$

The results at $T=0$ could be further simplified in the limit of large distances $d\to\infty$, where the exponents decouple (so they are independent of the distance d), and we consider the symmetric case $T_1=T_2$ where $S^{(0)}_{1\downarrow 2'\downarrow}=0$. We have in this limit

$$S^{d\to\infty}_{1\downarrow 2'\downarrow}(V) = S^{(\mathrm{int})d\to\infty}_{1\downarrow 2'\downarrow}(V) = +4\frac{e^2}{2\pi\hbar}R_ST_S\left(R_1T_1|eV|+\frac{\hbar}{2\pi}\times\int_{-\infty}^{\infty}\frac{dt}{t^2}\left(\left|\left\langle e^{-i\pi\mathcal{N}_{A_+}(t)}\right\rangle\left\langle e^{i\pi\mathcal{N}_{A_-}(t)}\right\rangle\right|^2-1\right)\right), \quad (39)$$

where the averages are taken with respect to the non-interacting Fermi seas transformed by the scattering matrix $\mathcal{S}_{1,2}$. Note first that in the case of $T_1 = T_2 = 1/2$ one can observe numerically that the averages are well-described by the analytical expression (see also the Supplementary Material of Ref. 1):

$$\left|\left\langle e^{i\pi\mathcal{N}_{A_\pm}(t)}\right\rangle\right|^2 = e^{-(eVt/4\hbar)^2}. \tag{40}$$

Using this expression (which seems to be exact, but we do not have a proof), one can write the noise in the following form

$$S^{d\to\infty}_{1\downarrow 2'\downarrow}(V, T_1 = T_2 = 1/2) = \frac{e^2}{2\pi\hbar} R_S T_S |eV| \left(1 - \sqrt{\frac{2}{\pi}}\right). \tag{41}$$

We note that the cross-correlations are positive, compared to zero cross-correlations in the case of $T_1 = T_2$ in the non-interacting limit.

2. *Dilute beam asymptotics*

In the case of dilute beams with equal transmission $T_1 = T_2 = T \to \{0, 1\}$ and at $d \to \infty$ we can use the asymptotics developed in the theory of FCS. At short times the product of the exponents in the Eq. (39) has a quadratic behaviour $1 - \alpha t^2$, where constant α depends on the tunneling, so the integral converges at short times. This contribution is trivial, and below we focus on the long-time asymptotics.

The long-time asymptotics can be calculated using the Fisher-Hartwig approach for the Toeplitz matrices developed in Ref. 5. Note that in the Supplemental Material of Ref. 1 regarding the equilibration of edge states, we showed a comparison of these asymptotics with the numerical results, and it was pointed out that the asymptotics break down at tunneling T=1/2 (see above).

Let us first reproduce the equations obtained in Ref. 5. We consider a double-step Fermi distribution at zero temperature

$$n(\varepsilon) = T\, n_0(\varepsilon - \mu_1) + R\, n_0(\varepsilon - \mu_2), \tag{42}$$

where $n_0(\varepsilon) = \theta(-\varepsilon)$ is a step function, $\mu_2 > \mu_1$ are the chemical potentials, $R = 1 - T$ is the reflection probability of the QPC, and $V = \mu_2 - \mu_1$ is bias voltage.

We further introduce the following constants, taking δ the fractionalization parameter in $\langle e^{-i\delta\hat{N}(t)}\rangle$ to be $\delta = \pi$ and assuming[6] $R < 1/2$

$$\beta_1 = -\frac{i}{2\pi}\ln(1 - 2R), \quad \beta_0 = -\frac{1}{2} - \beta_1, \tag{43}$$

as well as the dephasing time

$$t_\phi^{-1} = -\frac{eV}{2\pi\hbar}\ln(1 - 4RT). \tag{44}$$

With these definitions, the asymptotics of $\left\langle e^{-i\pi\hat{N}(t)}\right\rangle$ at long times (normalised to the equilibrium value), are given by the following expression obtained in Ref. 5

$$\Delta(t) = \left\langle e^{-i\pi\hat{N}(t)}\right\rangle_{\text{norm}} \sim e^{-t/2t_\phi}(Vt)^{\frac{1}{4}-\beta_0^2-\beta_1^2} = (1 - 4RT)^{eVt/4\pi\hbar}(Vt)^{\ln^2(1-2R)/2\pi^2}(Vt)^{i\ln(1-2R)/2\pi}. \tag{45}$$

Now let us use these expressions to calculate the noise. We need the absolute value of $\Delta(t)$, which reads

$$|\Delta(t)| \sim (1 - 4RT)^{eVt/4\pi\hbar}(Vt)^{\ln^2(1-2R)/2\pi^2}. \tag{46}$$

This function has an exponential decay times a power-law, and for small reflections $R \ll 1$ we get

$$|\Delta(t)| \sim (1 - 4R)^{eVt/4\pi\hbar}(Vt)^{2(R/\pi)^2}. \tag{47}$$

In order to obtain the tunneling-dependent contribution to the noise we need to integrate these asymptotics

$$\tilde{s}(V, R) = 2\int_\tau^\infty \frac{1}{t^2}(|\Delta(t)|^4 - 1)dt, \tag{48}$$

where we have introduced a cutoff τ. Note that if we do not assume the smallness of the reflection R, then we have to calculate the following integral:

$$\tilde{s}(V,R) = 2\int_{\tau}^{\infty} dt\ \frac{1}{t^2}\left((1-4RT)^{eVt/\pi\hbar}(eVt/\hbar)^{2\ln^2(1-2R)/\pi^2} - 1\right). \tag{49}$$

One can express this integral in terms of a special function (where we introduce a dimensionless cutoff $\Theta = eV\tau/\hbar$), but we only need its asymptotic development at small R, which gives

$$\begin{aligned} S_{1\downarrow 2'\downarrow}(V, T_1 = T_2)/(4R|eV|R_S T_S e^2/2\pi\hbar) \simeq \frac{4}{\pi^2}\ln(R) + 1 - \frac{4}{\pi^2}(\ln(4\Theta/\pi) + \gamma - 1) \\ + \frac{4}{\pi^2}R\ln(R) + R\left(-1 + \frac{8}{\pi^3}(1-\Theta) + \frac{4}{\pi^2}(\ln(4\Theta/\pi) + \gamma)\right), \end{aligned} \tag{50}$$

which has the most singular terms containing logarithms independent of the cutoff, as well as the constant term and the term proportional to R, which do depend on the cutoff. Using the structure of this expansion with respect to R, we can obtain the numerical values of the cutoff-dependent terms by fitting the numerical results obtained from the theory. This gives the following terms in the noise asymptotics at small R

$$S_{1\downarrow 2'\downarrow}(V,R)/(4R|eV|R_S T_S e^2/2\pi\hbar) \simeq \frac{4}{\pi^2}\ln(R) + \frac{4}{\pi^2}R\ln(R) - 0.2995R + 0.9427. \tag{51}$$

Finally, we can rewrite this in the notation used in the Main text in terms of the generalized Fano factor P and using the symmetry $\tau \to 1-\tau$:

$$P \simeq \frac{4}{\pi^2}\ln\tau_\mathrm{s} + \frac{4}{\pi^2}\tau_\mathrm{s}\ln\tau_\mathrm{s} - 0.2995\tau_\mathrm{s} + 0.9427. \tag{52}$$

For $\tau_\mathrm{s} = 0.05$ we obtain $P \simeq -0.35$, close to the experimental value in Fig. **3** of the Main text.

3. Finite temperature expressions

Here, we provide the finite temperature expression for the cross-correlation noise at zero frequency in the standard collider configuration shown Supplementary Fig. **1**. The following expression, obtained along the same lines described above for $T = 0$, was computed numerically for the data-theory comparison:

$$\begin{aligned} S_{1\downarrow 2'\downarrow}(V) = 2\frac{e^2}{2\pi\hbar}eVR_S T_S(R_1T_2 + T_1R_2)(\coth(eV/2k_BT) - 2k_BT/eV) \\ + 4\frac{e^2}{2\pi\hbar}eVR_S T_S(\pi k_BT/eV)^2\ \mathrm{Re}\int_{-\infty}^{\infty}\frac{d\tau}{2\pi\hbar}\ \sinh^{-2}(\pi k_BT\tau/\hbar)(K_{1\downarrow}(d,\tau)\bar{K}_{2'\downarrow}(d,\tau) - 1). \end{aligned} \tag{53}$$

We also provide the finite temperature expression for the cross-correlation noise at zero frequency in the alternative configuration where the analyzer QPC$_S$ is tuned to couple the two inner edge channels ($2'_\uparrow$ and $1_\uparrow$, which are not directly excited by the source QPC$_{1,2}$) with the transmission probability T_S:

$$S_{1\uparrow 2'\uparrow}(V) = +4\frac{e^2}{2\pi\hbar}eVR_S T_S(\pi k_BT/eV)^2\ \mathrm{Re}\int_{-\infty}^{\infty}\frac{d\tau}{2\pi\hbar}\ \sinh^{-2}(\pi k_BT\tau/\hbar)(K_{1\uparrow}(d,\tau)\bar{K}_{2'\uparrow}(d,\tau) - 1). \tag{54}$$

E. Electron energy distribution configuration

The same refermionization approach can be used to address the configuration implemented to observe the electron energy distribution. The different biasing corresponds to applying a second bias voltage V_p to both the edges 1 and 2 (following the labels in Supplementary Fig. **1**), which changes the chemical potentials provided by Eq. (26) for the collider configuration. In that case, QPC$_2$ does not play any role and the edges 1 and 2 can be merged into a single edge, as schematically pictured in Supplementary Fig. **2**.

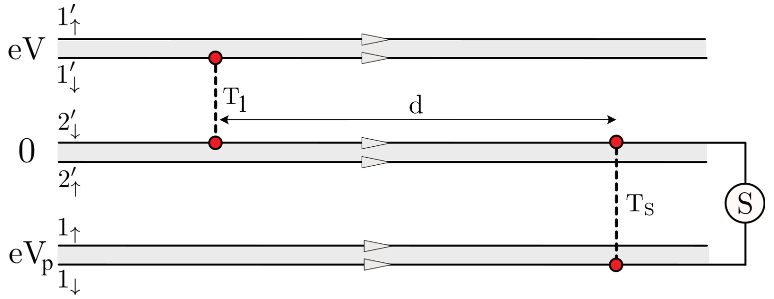

Supplementary Figure 2. Schematic of the configuration pertaining to the distribution measurement. The channels $1'_{\downarrow}$ and $2'_{\downarrow}$ are coupled via a source QPC positioned at $x = 0$ with the tunneling probability T_1. The channels $2'_{\downarrow}$ and $1_{\downarrow}$ are coupled by the analyzer QPC with tunneling probability T_S positioned at $x = d$, after which the current correlations are measured.

Along the same lines as in the collider configuration, the cross-correlations at zero-frequency and finite temperature T in the electron energy distribution configuration can be written:

$$S_{1\downarrow 2'\downarrow}(V, V_\mathrm{p}) = 4\frac{e^2}{2\pi\hbar} eV R_S T_S \times \left(\frac{k_B T}{eV} - \frac{V_\mathrm{p}}{2V} \tanh^{-1}\left(\frac{eV_\mathrm{p}}{2k_B T}\right) + \frac{eV}{2}\left(\frac{\pi k_B T}{eV}\right)^2 \mathrm{Re}\int_{-\infty}^{\infty} \frac{d\tau}{2\pi\hbar}\, e^{ieV_\mathrm{p}\tau/\hbar} \sinh^{-2}(\pi k_\mathrm{B} T\tau/\hbar)(\bar{K}_{2'\downarrow}(d,\tau) - 1)\right), \quad (55)$$

where the channels A'_+ and A'_- are set to voltage V and 0, respectively and a similar expression for the measurement in channel $2'\uparrow$ with the function $K_{2'\uparrow}(d, t)$. The numerical calculations of S_{12} based on Eq. (55) are compared to the data in Fig. **2** in the Main text, as well as in Supplementary Figs. **5**,**6**,**7**.

II. ANYONIC EXCHANGE THEORY

The left and right source QPC inject electrons towards the central analyzer where cross-correlations are measured. Because of interchannel interaction, the electron wave-packets of charge e separate into twin wave-packets carrying each half of the electron charge $e/2$. They correspond to the neutral and charge modes of the two copropagating channels progressing with distinct velocities v_n and $v_\mathrm{c} > v_\mathrm{n}$. The resulting beam has a mixed nature: the injection is random (and poissonian for $\tau_\mathrm{s} \ll 1$) for the center of mass of the twin wave-packets, whereas the distance between them is purely deterministic.

An alternative way of producing fractional charges in the integer quantum Hall case was proposed in Ref. 7 by using a metallic quantum dot (QD) as a source. Only the neutral mode is then excited and the train of charge $e/2$ is predicted to be entirely randomly distributed. Using a fully quantum bosonization approach, the cross-correlations out of an analyzer QPC were computed[7] to be

$$S_{12} \sim \tau_\mathrm{c}\, S_\Sigma^{QD}\, \frac{2\,(\sin\theta)^2}{\theta^2} \ln \tau_\mathrm{s} \quad (56)$$

in the balanced case with two sources, $\tau_\mathrm{s} \ll 1$, and with τ_c the analyzer transmission. $\theta = \pi/2$ is the mutual exchange phase between an electron and a fractional charge $e/2$. This results from the partition noise of the analyzer by electron-hole pair creation yielding a negative contribution. Remarkably, the partition term was identified[7,8] to be the consequence of a braiding mechanism in 1+1 (space and time) dimension involving the fractional excitation encircling the path of the electron-hole pair.

At low temperature, the noise of the sources is mostly shot noise, proportional to the granular charge of the signal. This is e for our geometry as electrons tunnel from the source QPC whereas it is $e/2$ with the metallic quantum dot of Ref. 7. Therefore, the incoming shot noise is twice larger in our experiment, $S_\Sigma = 2S_\Sigma^{QD}$, for the same quasiparticle current. Normalizing the cross-correlation of Eq. (56) with $\tau_\mathrm{c} S_\Sigma$ (see Eq. (2) in the Main text), we find for the partition noise $P = (4/\pi^2)\ln\tau_s$, exactly as the leading term in Eq. (52) derived within the complete non-perturbative theory applied to our geometry. We can draw two consequences from this result:

1. the non-perturbative theory is in excellent agreement with our experimental data. Although the braiding mechanism is not transparent in this theory, the exact asymptotic matching in the dilute limit $\tau_\mathrm{s} \ll 1$ with the

braiding theory of Ref. 7 shows that this theory encompasses the braiding process. The partition noise is the same in Ref. 7 and in our experiment.

2. our experiment nonetheless differs from the quantum dot fractionalization proposal in that the noise generated by the sources is two times larger. We thus need to multiply the first term of Eq. (56) by a factor 2 and obtain

$$P = \frac{S_{12}}{\tau_c S_\Sigma} \sim \frac{(\sin\theta)^2}{\theta^2} \ln \tau_s = \frac{4}{\pi^2} \ln \tau_s. \tag{57}$$

III. RANDOM OR DETERMINISTIC INJECTED SIGNAL

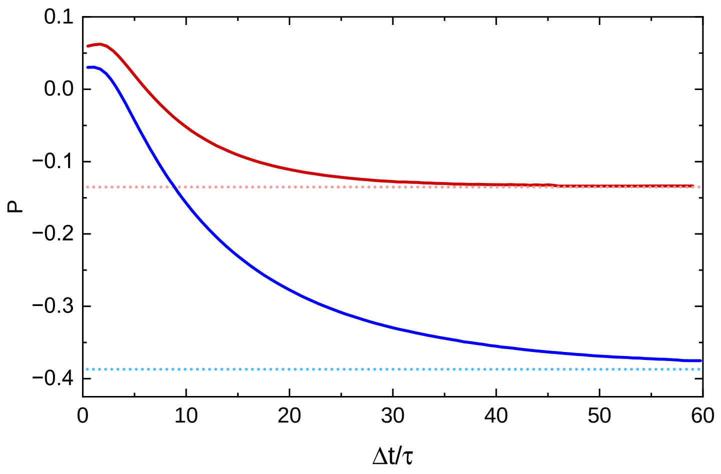

Supplementary Figure 3. Cross-correlations P (plain lines) as function of the time delay Δt between the twin pulses. The dotted lines give P for random $e/2$ pulses. $\tau/\tau_s = 0.005$ and $\tau/\tau_s = 0.01$ are shown in red and blue.

We turn here to the issue of random vs deterministic for the injected beam since the distance between twin pulses is fixed. The asymptotic matching of the non-perturbative theory with the theory of Ref. 7, where the injection is random, suggests that the distinction is not important as long as the time splitting between twin wave-packets exceeds largely the mean distance between subsequent pairs. Ref. 8 has shown that the injection by the source quantum point contacts, as far as cross-correlations are concerned, is generally equivalent to the driving by an ohmic contact with a series of random quantized voltage pulses. We thus consider here a series of twin pulses, with time splitting Δt, sent with a Poissonian distribution. Following Ref. 8, we use the Kubo formula and obtain for the renormalized cross-correlation noise

$$P = -\frac{1}{4\pi^2} \int_{-\infty}^{+\infty} dt \frac{e^{-\frac{\tau_s}{\tau}(g(t)+g^*(t))}}{(0^+ + it)^2}, \tag{58}$$

where τ is the temporal width of individual pulses. We have introduced the function

$$g(t) = \int_{-\infty}^{+\infty} dt' \left(1 - e^{i\left(\phi_0(t-t') - \phi_0(-t')\right)}\right), \tag{59}$$

where, for twin Lorentzian pulses of charge $e/2$,

$$\phi_0(t) = \frac{e}{\hbar} \int_{-\infty}^{t} dt'\, V_0(t') \qquad V_0(t) = \frac{h}{2e} \left(\frac{\tau/\pi}{(t-\Delta t/2)^2 + \tau^2} + \frac{\tau/\pi}{(t+\Delta t/2)^2 + \tau^2} \right). \tag{60}$$

Supplementary Fig. **3** illustrates the comparison of this calculation with a direct evaluation of P for purely random pulses (and not series of twin pulses) of charges $e/2$, confirming that they coincide in the limit of very long time delay $\Delta t \gg \tau/\tau_s$.

IV. PHENOMENOLOGICAL FERMIONIC THEORY

Here we consider an alternative model which also yields negative cross-correlations in the dilute limit. It is a phenomenological fermionic model which takes interactions into account via energy redistribution into hot Fermi distribution functions.

In the collider geometry discussed in the Main text, we consider a channel which starts out with a double-step initial distribution, and following inter-channel energy transfer it has the relaxed Fermi distribution with an effective temperature

$$T^* = \sqrt{T^2 + \frac{3}{2}\tau_\mathrm{s}(1-\tau_\mathrm{s})\left(\frac{eV_\mathrm{s}}{\pi k_\mathrm{B}}\right)^2}, \tag{61}$$

where V_s is bias, T is base temperature, and τ_s is the transmission of the source QPC[9,10].

This kind of phenomenological description has previously been used for explaining experimental results (see, e.g., Ref. 11 at $\nu = 1$ and Refs. 9,10,12 at $\nu = 2$). The cross-correlations have the form:

$$S_{12} = \tau_\mathrm{c}(1-\tau_\mathrm{c})S_\Sigma - 2\frac{e^2}{h}\tau_\mathrm{c}(1-\tau_\mathrm{c})\int_{-\infty}^{\infty}\left(f_\mathrm{L}(1-f_\mathrm{R}) + f_\mathrm{R}(1-f_\mathrm{L})\right)d\varepsilon, \tag{62}$$

with L (R) denoting the left (right) distribution in the outer channel and S_Σ the total noise incoming from both sources which is equal to $2\times 2\frac{e^2}{h}\tau_\mathrm{s}(1-\tau_\mathrm{s})eV_\mathrm{s}\left(\coth\frac{eV_\mathrm{s}}{2k_\mathrm{B}T} - \frac{2k_\mathrm{B}T}{eV_\mathrm{s}}\right)$ in the case of balanced beams. Plugging the Fermi distribution with temperature from Eq. (61) into Eq. (62), we find

$$S_{12} = \tau_\mathrm{c}(1-\tau_\mathrm{c})S_\Sigma - 4\frac{e^2}{h}\tau_\mathrm{c}(1-\tau_\mathrm{c})k_\mathrm{B}T^*, \tag{63}$$

which at $T = 0$ yields the Fano factor

$$P = 1 - \frac{\sqrt{3}}{\pi\sqrt{2\tau_\mathrm{s}(1-\tau_\mathrm{s})}}. \tag{64}$$

Therefore, this alternative description of the system with interacting fermions can also lead to non-zero cross-correlations with the same change of sign between the dilute regime and the $\tau_\mathrm{s} \sim 0.5$ regime, due to the relative importance of the positive source noise redistribution and negative cross-correlation contribution. Moreover, as seen in Supplementary Fig. **4**, the shape of the prediction for both models is similar, although the quantitative values are different. We therefore conclude that the mere presence of negative cross-correlations cannot be directly attributed to the anyonic exchange phase, since a simple fermion model also qualitatively predicts it. In order to be able to attribute the negative signal to the fractional statistics of the involved charges, we need to validate the non-perturbative approach by complementary distribution measurements, as we have done in the Main text. Note that we cannot directly rule out the phenomenological fermionic theory because this would require one to compare the observed distributions with specific predictions. However this phenomenological theory does not allow one to make specific predictions regarding the evolution of the distribution as this would require to introduce a choice for the rate of inelastic collisions as a function of the exchanged energy.

V. FITTING PROCEDURE TO DETERMINE δt

We outline the procedure used to obtain the only fitting parameter of the theory, the time delay δt between the arrival of fractionalized $e/2$ charges at the analyzer QPC, namely $\delta t = d/v_\mathrm{n} - d/v_\mathrm{c}$, with d the distance between source and analyzer quantum point contacts and $v_\mathrm{c,n}$ the velocities of charged and neutral mode.

In the source-analyzer configuration we measure the cross-correlations $S_{12}(V_\mathrm{p})$ which yield the distributions $f(\varepsilon = eV_\mathrm{p})$ (Main text Eq. (1)). Cross-correlations $S_{12}(V_\mathrm{p})$ contain a big contribution from the equilibrium noise :

$$S_{12}{}^0 = 2\frac{e^2}{h}\tau_\mathrm{s}(1-\tau_\mathrm{s})|eV_\mathrm{p}|\left(\coth\left(\frac{eV_\mathrm{p}}{2k_\mathrm{B}T}\right) - \frac{2k_\mathrm{B}T}{eV_\mathrm{p}}\right). \tag{65}$$

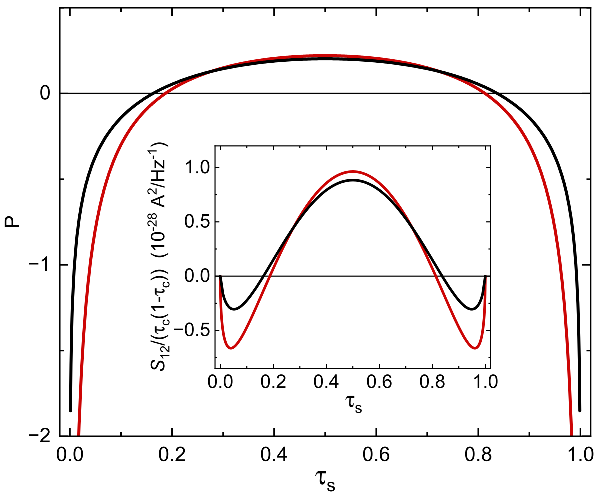

Supplementary Figure 4. **Comparison between the phenomenological and the non-perturbative framework.** Inset: Renormalized cross-correlations $S_{12}/(\tau_c(1-\tau_c))$ versus the source transmission τ_s are shown at $T = 0$ K. The black line is the prediction of the non-perturbative theory, and the red line is the prediction of the phenomenological fermionic theory. Main panel: Fano factor P calculated in the two approaches.

After subtraction we obtain $dS_{12}(V_p) = S_{12}(V_p) - {S_{12}}^0(V_p)$, which is very sensitive to the value of δt. We find the best value by a least-squares method in the data subset corresponding to the [59 µV,82 µV] range of bias voltage. We chose this range because it corresponds to the regime of fully fractionalized charge. As given in the Main text, we have determined $\delta t = 64$ ps. With $d = 3.1$ µm measured in the SEM photo (Fig.**1**c, Main text), we obtain $d/\delta t = 5 \times 10^4$ m s^{-1}. We find slightly different values of δt for left and right side, namely $\delta t_L = 68 \pm 2$ ps and $\delta t_R = 60 \pm 2$ ps. If we assume that the velocity difference between the fast and slow mode is the same on both sides, this yields the left and right distance of 3.3 ± 0.1µm and 2.9 ± 0.1µm. This is plausible if we consider the SEM photo which reveals a slightly longer distance between the source and analyzer QPC on the right-hand side (Fig. **1**c, Main text). The difference can also originate from the screening details in the edge. However, since the theory is developed for equal lengths on the left and right, we adopt the mean value of $\delta t = 64$ ps which we use throughout.

VI. DISTRIBUTIONS

A. Distributions at intermediate bias voltages

In Supplementary Fig. **5** we expand on the data shown in Fig. **2** of the Main text and show the full evolution of the distribution function for bias voltages between 12 µV and 82 µV. The injection and measurement take place on the outer channel. We see the relaxation from the double-step at lower bias into a single broader-step distribution in the range 47-70 µV.

At $V_s = 82$ µV we have some inter-channel tunneling starting to take place, see Section XI below.

B. Distributions at low transparency

In order to verify that the charge fractionalization in the dilute limit does not deviate from the expected behavior, we measured the distributions at source transmission $\tau_s = 0.05$ and $\tau_s = 0.95$, shown in Supplementary Fig. **6**. In the inset we show S_{12} used to obtain $f(\varepsilon)$ by derivation, see Eq. (1) in the Main text.

We see that the finite- and infinite-time predictions (purple and blue curve) are very close, and explain the data very well. With the same reasoning outlined in the Main text, from this we conclude that the full fractionalization has taken place for dilute beams. We shall use this result for the cross-correlation 'collider' measurement.

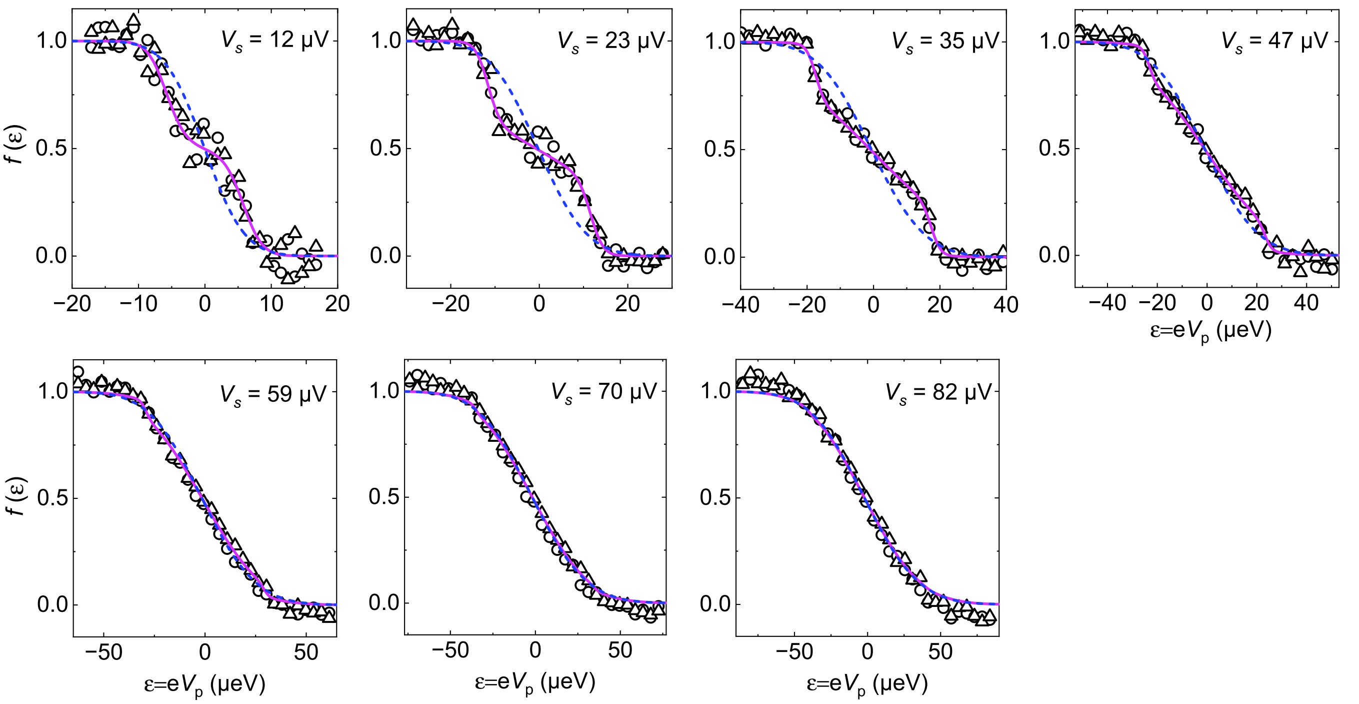

Supplementary Figure 5. **Electron energy distribution spectroscopy at intermediate voltages.** Electron energy distribution f vs injected energy ε for the source bias voltage V_s given in each panel, ranging from 12 μV to 82 μV. The additional V_s complete the three values shown in Fig. **2** in the Main text. Squares and triangles correspond to V_s applied, respectively, to the left and right source QPC. Both source and analyzer transmissions are set to 0.5. Continuous purple lines show the theoretical predictions for $\delta t = 64$ ps and the dashed blue lines those for $\delta t = \infty$.

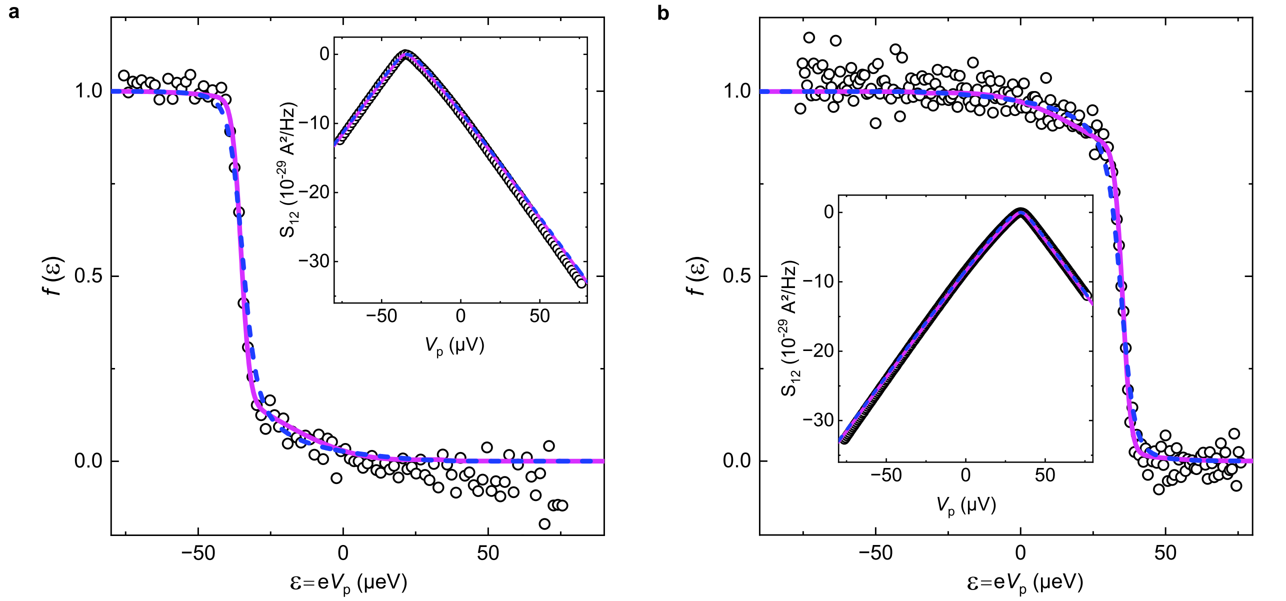

Supplementary Figure 6. **Electron energy distribution spectroscopy at high beam dilution.** Energy distribution f obtained from S_{12} (insets) by derivation, see Main text, Eq. (1). Panel **a** corresponds to $\tau_s = 0.05$ and panel **b** to $\tau_s = 0.95$. The analyzer transmission is set to $\tau_c = 0.5$ and bias voltage to $V_s = 70$ μV. Injection is from the right QPC. Full purple and dashed blue line are the predictions for $\delta t = 64$ ps and $\delta t = \infty$ respectively.

C. Inner channel distributions

In Supplementary Fig. **7** we show the measured distributions in the configuration where we inject into the inner channel, and measure on the outer, see the schematic in the inset. In this case we don't see the double-step at low bias, since there was no injection, but we see the broadening of the initial distribution when increasing bias. At full relaxation we expect the distribution on the injection channel to match that on the other channel. Theoretically this is the case (purple and blue curve compared to their counterpart in Supplementary Fig. **5**), but experimentally we see that the relaxation is not complete.

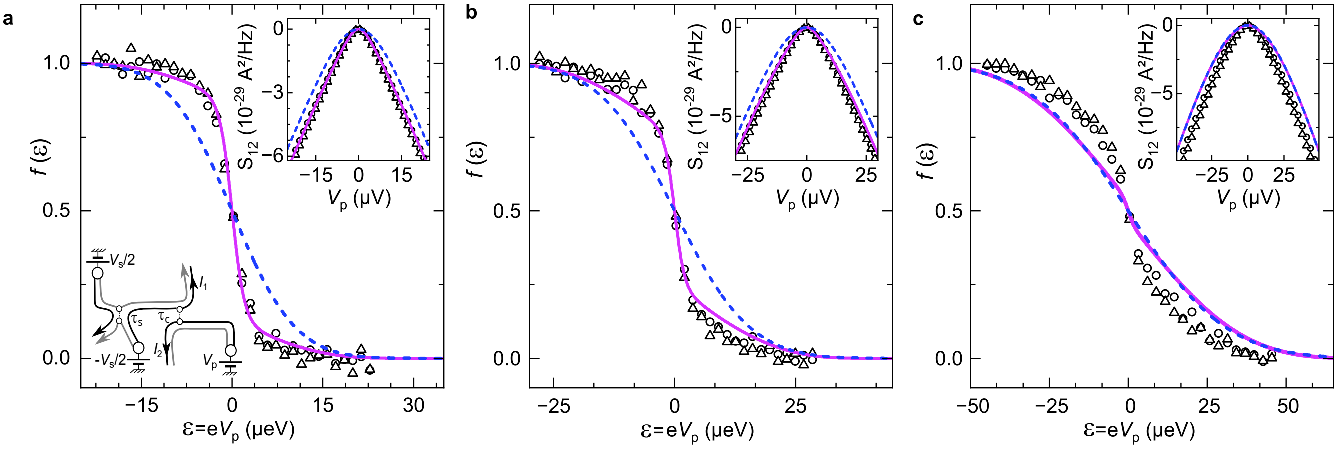

Supplementary Figure 7. **Electron energy distribution spectroscopy along the inner edge channel. a,b,c** Energy distribution $f(\varepsilon)$ obtained by deriving the measured S_{12} (insets, see Eq. (1) and plotted versus the probe energy $\varepsilon = eV_\mathrm{p}$, taken at bias voltage V_s = 23 µV, 35 µV and 70 µV from left to right. The injection is on the inner channel and the measurement on the outer (see schematic in **a**). Circles and triangles correspond respectively to the injection from the left and right source. Both source and analyzer transmissions are tuned to $\tau_\mathrm{s} = \tau_\mathrm{c} = 0.5$. Full purple and dashed blue line display the numerical predictions for δt = 64 ps and $\delta t = \infty$ respectively.

VII. CROSS-CORRELATIONS FOR A DILUTE VS NON-DILUTE BEAM

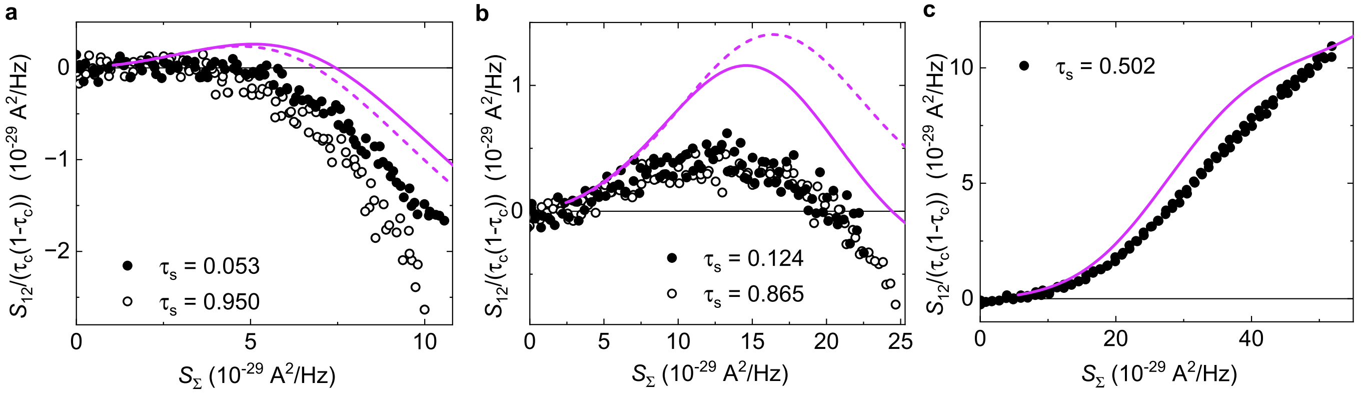

Supplementary Figure 8. **a, b, c,** Cross-correlations $S_{12}/(\tau_\mathrm{c}(1-\tau_\mathrm{c}))$ vs source noise S_Σ at transparencies $\tau_\mathrm{s} = 0.053/0.95$ (**a**), 0.124/0.865 (**b**) and 0.502 (**c**). All quantum point contacts are tuned to outer channels. In panels **a** and **b** full circles denote data taken at low transparency τ_s, and open circles at transparency close to 1. The purple lines denote the prediction for δt = 64 ps, full lines for $\tau_\mathrm{s} = 0.053/0.124$ and dashed lines for $\tau_\mathrm{s} = 0.95/0.865$ in **a**/**b** respectively.

In Supplementary Figure **8** we show cross-correlations $S_{12}/(\tau_\mathrm{c}(1-\tau_\mathrm{c}))$ vs S_Σ in three regimes when they are negative, at the transition, and positive. In Supplementary Fig. **8**a we are interested in the low transparency $\tau_\mathrm{s} = 0.053$ (full circles) and its complement $\tau_\mathrm{s} = 0.95$ (open circles) (same data as in Main text Fig. **3**) . In Supplementary Fig. **8**b at $\tau_\mathrm{s} = 0.124, 0.865$ (full/open circles respectively) the system is in the intermediate regime where the cross-correlations

change sign. In Supplementary Fig. **8**c taken at $\tau_s = 0.502$, the cross-correlations are positive in the full bias range. The purple curves are the prediction of the non-perturbative theory for $\delta t = 64$ ps. Full curves are predictions for $\tau_s = 0.053/0.124$ and dashed curves for $\tau_s = 0.95/0.865$. There is a slight difference between the full and dashed curve in each panel because τ_s and $1 - \tau_s$ are not exactly the same. We see that the slopes are reasonably well reproduced by the theory, but the detailed agreement at low bias is absent. The prediction around zero bias is not available as it requires very long calculation times.

Supplementary Fig **8**a shows the same data as Fig. **3** in the Main text for $\tau_s = 0.053/0.95$. The two datasets should yield a similar slope, i.e., a similar Fano factor. We notice that the experimental Fano factor is slightly different in the two cases. The slope of the negative part is $P \simeq -0.38$ at $\tau_s \sim 0.05$, and $P \simeq -0.56$ at $\tau_s \sim 0.95$. Some part of the discrepancy may be due to the tunneling between copropagating channels which starts to appear at large voltage (see Supplementary Figs. **12** and **13** and the tunneling discussion below), or to the experimental particularities such as the nonequivalent paths towards the analyzer or the temperature difference between the ohmic contacts ~ 1 mK (see Methods). We also see asymmetry between τ and $1 - \tau$ in Fig. **4** in the Main text, and in Supplementary Fig. **10**.

The uncertainties on measured source QPC transparencies are the following : 0.053 ± 0.002, 0.950 ± 0.001, 0.124 ± 0.004, 0.865 ± 0.003 and 0.502 ± 0.004. We are using the average of the high-bias region where the transmission dependence on bias is weak (cf. Fig **3**a in the Main text).

VIII. CROSS-CORRELATIONS IN THE INNER CHANNEL

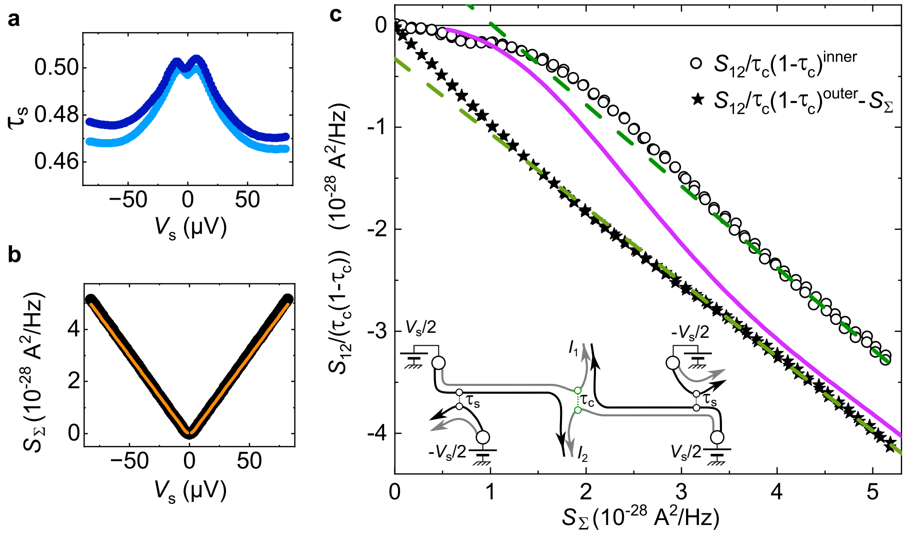

Supplementary Figure 9. **Cross-correlations with the analyzer QPC probing the inner channel. a**, Measured left/right source QPC dc transmission as a function of bias voltage, shown in light/dark blue, respectively. **b**, Source noise S_Σ vs bias voltage. The orange line displays Eq. (3) from the Main text with T =11 mK. **c**, Excess shot noise $S_{12}/(\tau_c(1-\tau_c))$ plotted versus the source noise S_Σ. Open symbols correspond to the measurement when the analyzer probes the inner channel. Full stars show $S_{12}/(\tau_c(1-\tau_c)) - S_\Sigma$ when the analyzer probes the outer channel. This data is the same as Supplementary Fig. **8**c, also for $\tau_s = 0.5$. The two are expected to be the same. The continuous purple line displays the theory for $\delta t = 64$ ps. Fits of the data in the region $V_s > 59$ µV (green dashed lines) give the Fano factors of 0.80 (inner channel) and 0.73 (outer channel).

In Supplementary Fig. **9** we show the cross-correlations when the charge is injected into the outer channels and the signal is measured in the inner channel. In that case, the positive contribution of the source noise is absent, and the signal is negative throughout. Like in the Main text, we show in Supplementary Fig. **9**a the transparencies of all quantum point contacts as function of bias, and in Supplementary Fig. **9**b that the source noise corresponds to the injection of charge e (slope of the orange fit). We see a higher variation in the central QPC transparency, but this transparency is not expected to affect the signal (we will demonstrate this in Supplementary Fig. **11**). In Supplementary Fig. **9**c we show the cross-correlations of the inner channel, and the cross-correlations of the outer channel with the source noise subtracted. These are expected to coincide. They do show roughly the same slope (green lines), but there is some discrepancy between the curves themselves. We do not understand this discrepancy beyond earlier observations that the injection into the inner channel was not well controlled.

IX. CROSS-CORRELATIONS IN THE FULL BIAS AND TEMPERATURE RANGE

In Supplementary Fig. **10** we show the cross-correlations as function of source transparency in the full bias range and for the additional temperature of 21 mK. As in Fig. **4** in the Main text the left/right column corresponds to the analyzer set to the outer/inner channel, respectively.

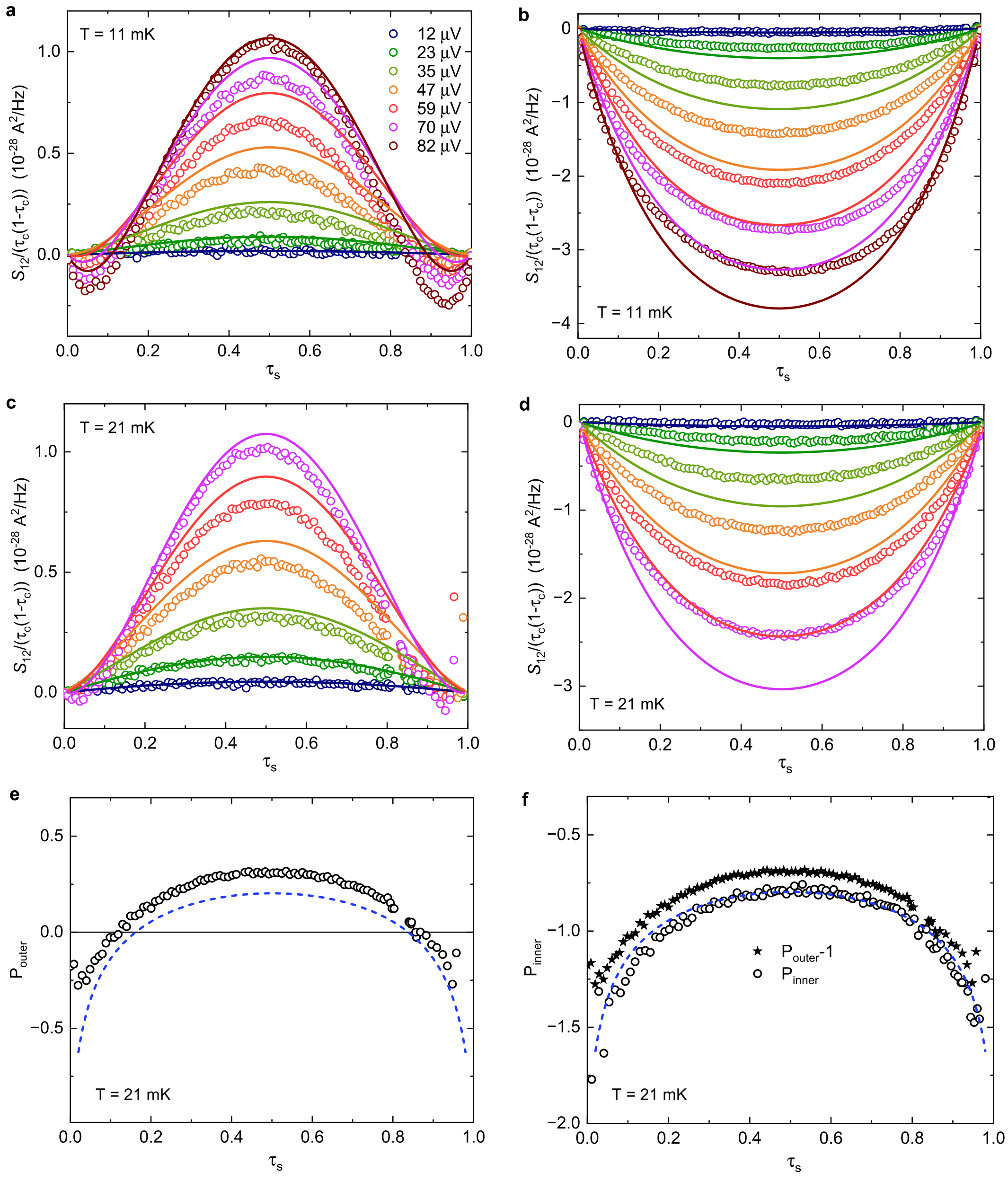

Supplementary Figure 10. **Cross-correlations vs dilution at intermediate voltages and higher temperature. a, c,** Cross-correlations $S_{12}/(\tau_c(1-\tau_c))$ as function of source QPC transmission τ_s at 11 mK (**a**,**b**) and 21 mK (**c**, **d**). Extension of Fig. **4** from the Main text. Injection takes place in the external channel at all times, whereas the measurement is done on the external channel (**a**, **c**, **e**) or the internal channel (**b**, **d**, **f**). The solid lines in **a-d** are the prediction for $\delta t = 64$ ps. Data at each bias voltage and their prediction have the same color (cf. legend). **e, f,** Fano factors extracted at $T = 21$ mK with the central QPC partially transmitting the outer (**e**) and inner (**f**) channel. Blue lines correspond to the high bias/large δt prediction. Full stars in panel **f** display $P_{\mathrm{outer}} - 1$.

X. CROSS-CORRELATIONS ARE INDEPENDENT OF THE ANALYZER TRANSMISSION

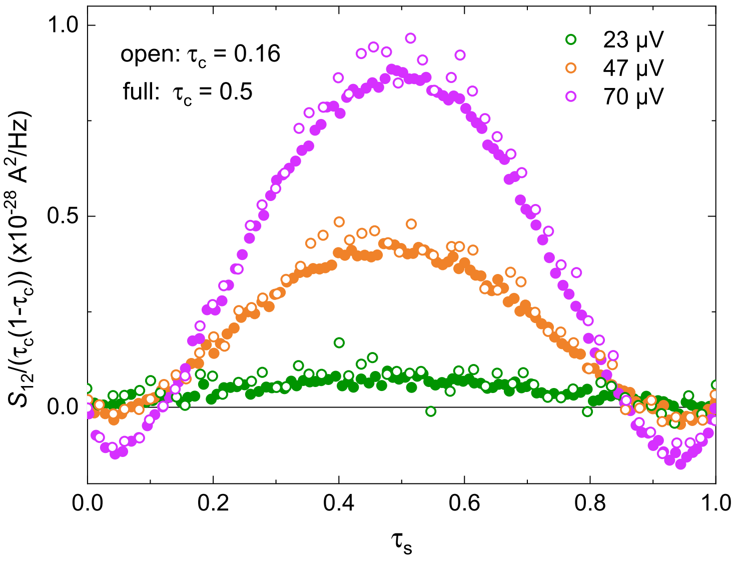

Supplementary Figure 11. Cross-correlations $S_{12}/(\tau_c(1-\tau_c))$ as function of source QPC transmission τ_s for $\tau_c = 0.5$ (open symbols) and $\tau_c = 0.16$ (full symbols). Bias voltages are shown in the figure.

As mentioned, the cross-correlation signal $S_{12}/(\tau_c(1-\tau_c))$ does not depend on the transparency of the analyzer. We have checked this by comparing the curves measured at $\tau_c = 0.5$ and $\tau_c = 0.16$ at three bias voltages, see Supplementary Fig. **11**. As another control measurement, we have verified that there is no cross-correlation signal when the analyzer QPC is set to transmission $\tau_c = 1$, i.e., is on the plateau. This is expected as there is no partition at the analyzer QPC in that case.

XI. TUNNELING BETWEEN THE INNER AND OUTER CHANNEL

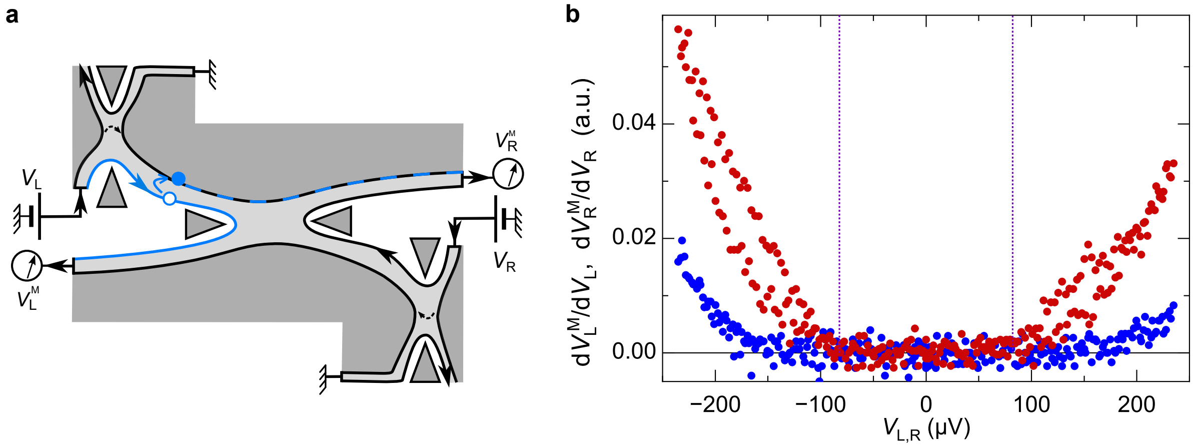

Supplementary Figure 12. **a**, Schematics of the tunneling process and the measurement setup. **b**, Measured differential conductance due to tunneling as function of bias voltage. Blue and red dots correspond to the injection from the left/right side.

At $\nu = 2$, tunneling between the two adjacent copropagating channels is usually negligible. However, at long effective propagating distance, tunneling events can develop and alter our cross-correlation signal. Indeed, a carrier hopping from the outer channel to the inner one results in current fluctuation δI on the inner channel and a correlated $-\delta I$

one in the outer channel. Therefore, such artifacts would create unwanted additional noise $-\tau_c^2 \delta I^2$ on the measured cross-correlations on the leads downstream to the central QPC.

We calibrate the tunneling by injecting energy on the outer channel while the central QPC is set on the plateau. In that configuration the only contribution to the signal is expected to come from tunneling. Therefore, measuring voltage $V^M_{\mathrm{R(L)}}$ at the frequency of $V_{\mathrm{L(R)}}$ can be directly attributed to the tunneling events along the path between the left (right) source and central QPC (see Supplementary Fig. **12**a). The fraction of current $dV^M_{\mathrm{R(L)}}/dV_{\mathrm{L(R)}}$ that tunnels between the edges is plotted in Supplementary Fig. **12**b as function of V. It is found to remain negligible in the bias range used for the main measurements $V \leq 82$ μV, indicated by purple vertical lines. Note that a hysteresis appears at higher voltage, prompting us to remain in the range $V \in [-82, 82]$ μV.

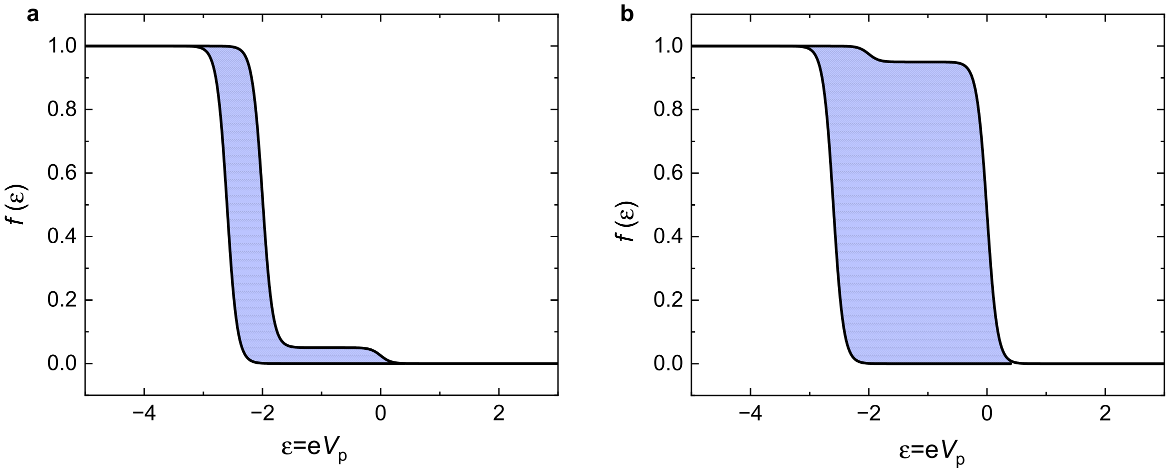

Supplementary Figure 13. Assumed distribution functions between two adjacent edge channels after the injection of a quasiparticle into one of them. The available tunneling phase space from one to the other channel is shaded. Panel **a/b** refers to the source transparency $\tau_s = 0.05/0.95$ respectively.

Another effect of tunneling considered in the Main text is it being a potential cause of asymmetry in the measured cross-correlations at $\tau_s = 0.05$ and 0.95. If we consider the two distribution functions in the two copropagating edge channels after the injection of the quasiparticle into one channel, we get the situation shown in Supplementary Fig. **13** where the channel with injection is a double-step function, and the adjacent channel is a single-step function. The phase space for tunneling from one channel to the other is shaded. As we see, it is much larger for $\tau_s = 0.95$, resulting in extra negative cross-correlation signal, consistent with our observations.

XII. OSCILLATIONS

The non-perturbative theory prediction shows oscillations with $(\delta t)^{-1}$ which are not found in the measurement. We remind that $\delta t = d/v_n - d/v_c$ is the time delay between fractionalized wave-packets, with d the distance between source and analyzer quantum point contacts and $v_{c,n}$ the velocities of charged and neutral mode. Moreover, δt is the only fitting parameter. In Supplementary Fig. **14** we numerically go to much higher bias than available experimentally in order to explore the asymptotic behavior. We expect the oscillations to dampen with bias and at sufficiently high bias to not have a difference between finite and infinite δt.

This is indeed what we find. The finite δt curves (black) oscillate above (low transmission) or under (high transmission) the corresponding $\delta t = \infty$ curves (blue), and, at high enough bias the black and blue curve coincide. Since the slope is the Fano factor (up to a multiplicative constant), we conclude that it should be calculated at $\delta t = \infty$.

We assume that the oscillations are due to cutoffs at finite energies $k_B T$ and $h/\delta t$. The discrepancy between data and theory in the collider geometry (as opposed to distributions) may be at least partially due to these oscillations.

XIII. LIMITATIONS OF THE NON-PERTURBATIVE MODEL

We note that some experimental details are beyond the scope of the model. The model does not include long-range

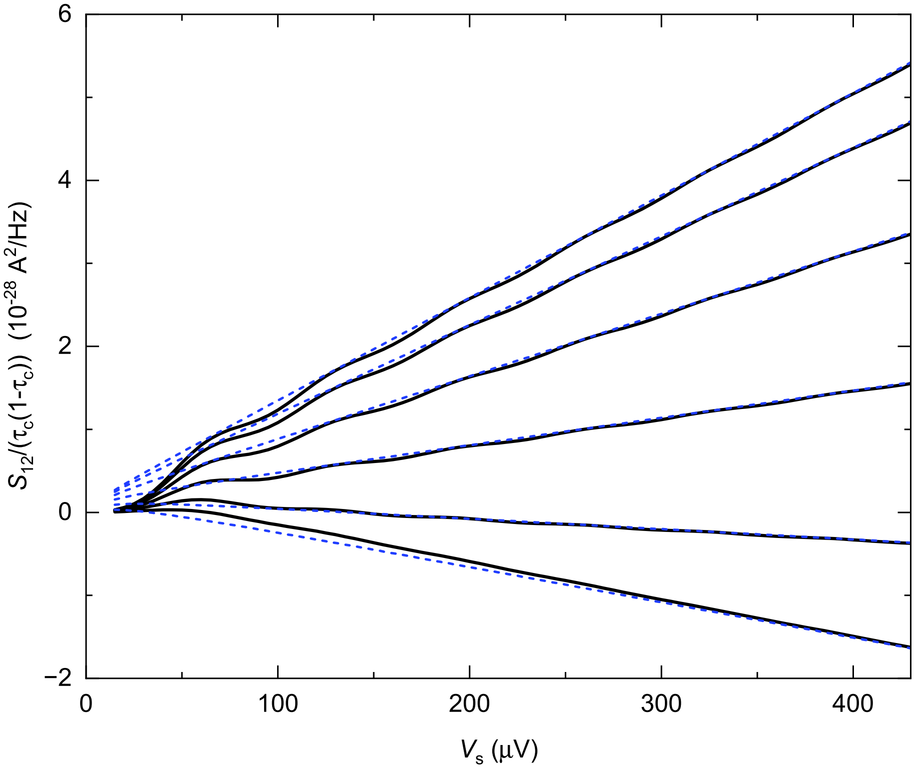

Supplementary Figure 14. Comparison of calculated cross-correlations for $\delta t = 64$ ps (black curves) and ∞ (blue curves). Transmissions τ_s range from 0.06 to 0.46 in steps of 0.08 (bottom to top).

interactions, dissipation, plasmon dispersion, or the coupling of the plasmon modes to the adjacent charge puddles.

---

[1] Kovrizhin, D. L. & Chalker, J. T. Relaxation in driven integer quantum Hall edge states. *Phys. Rev. Lett.* **109**, 106403 (2012).

[2] Kovrizhin, D. L. & Chalker, J. T. Multiparticle interference in electronic Mach-Zehnder interferometers. *Phys. Rev. B* **81**, 155318 (2010).

[3] Kovrizhin, D. L. & Chalker, J. T. Equilibration of integer quantum Hall edge states. *Phys. Rev. B* **84**, 085105 (2011).

[4] Rufino, M. J., Kovrizhin, D. L. & Chalker, J. T. Solution of a model for the two-channel electronic Mach-Zehnder interferometer. *Phys. Rev. B* **87**, 045120 (2013).

[5] Gutman, D. B., Gefen, Y. & Mirlin, A. D. Non-equilibrium 1d many-body problems and asymptotic properties of Toeplitz determinants. *J. Phys. A Mat. Theor.* **44**, 165003 (2011).

[6] Due to the symmetry between $S_{12}(T)$ and $S_{12}(1-T)$ we can have dilute beams for $T \to 1$ at the same time as $R < 1/2$.

[7] Morel, T., Lee, J.-Y. M., Sim, H.-S. & Mora, C. Fractionalization and anyonic statistics in the integer quantum Hall collider. *Phys. Rev. B* **105**, 075433 (2022).

[8] Mora, C. Anyonic exchange in a beam splitter (2022). ArXiv:2212.05123.

[9] le Sueur, H. *et al.* Energy relaxation in the integer quantum Hall regime. *Phys. Rev. Lett.* **105**, 056803 (2010).

[10] Degiovanni, P. *et al.* Plasmon scattering approach to energy exchange and high-frequency noise in $\nu = 2$ quantum Hall edge channels. *Phys. Rev. B* **81**, 121302 (2010).

[11] Ota, T., Hashisaka, M., Muraki, K. & Fujisawa, T. Negative and positive cross-correlations of current noises in quantum Hall edge channels at bulk filling factor. *J. Phys. Condens.* **29**, 225302 (2017).

[12] Altimiras, C. *et al.* Non-equilibrium edge channel spectroscopy in the integer quantum Hall regime. *Nat. Phys.* **6**, 34 (2010).
